# Supplementary material for: Structural basis for catalysis of human choline/ethanolamine phosphotransferase 1
Source: Nat Commun. 2023 May 3;14:2529. doi: 10.1038/s41467-023-38290-2 (PMC10156783; doi:10.1038/s41467-023-38290-2)
Supplement: Supplementary file 1 — Supplementary Information [file 41467_2023_38290_MOESM1_ESM.pdf]

Legends for Supplementary Data

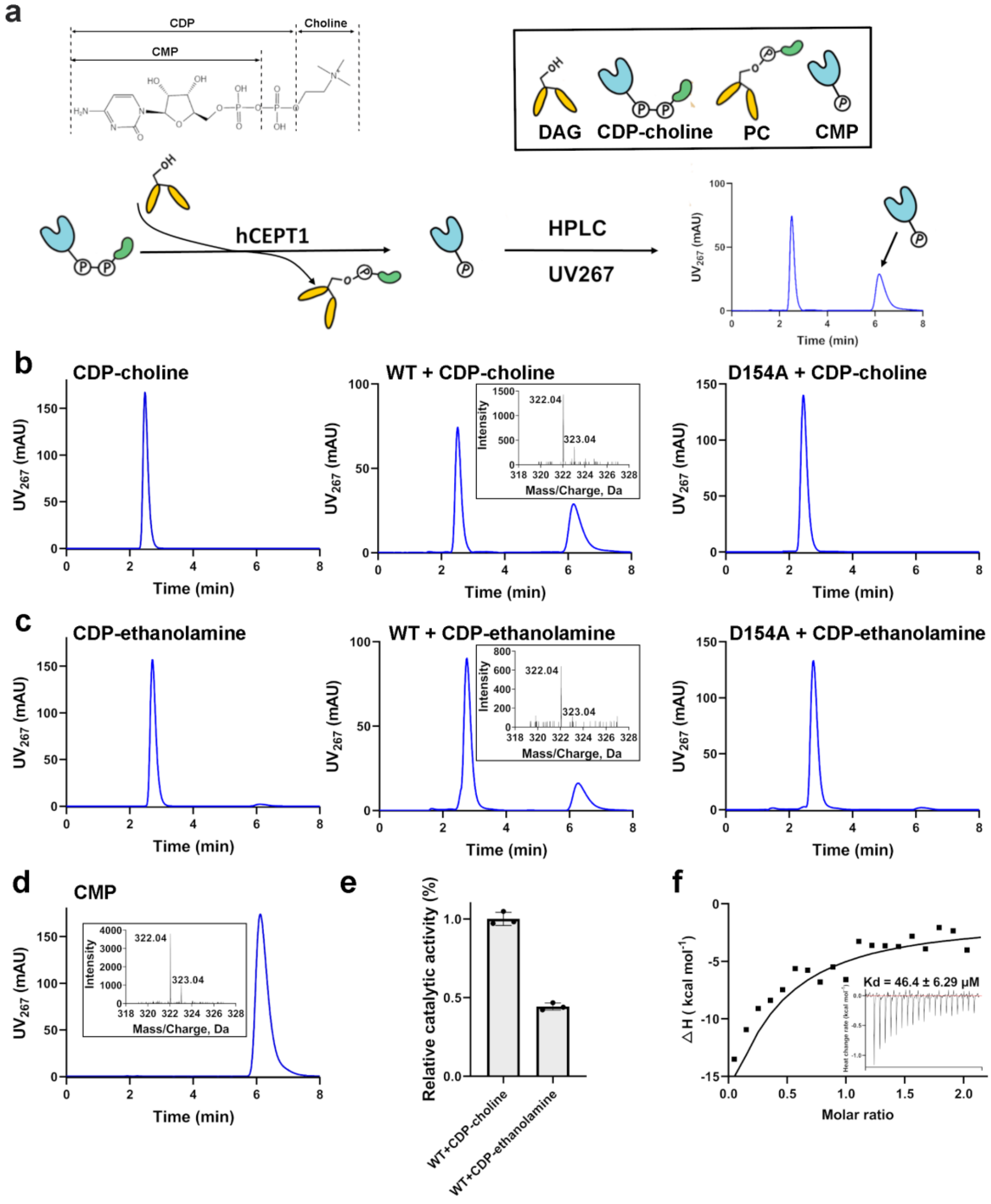

Supplementary Figure 1 | Enzymatic activity of purified human CEPT1. a,

Schematic diagram of our enzymatic assay for hCEPT1. The chemical structure of CDP-choline (top) and a schematic illustration of the HPLC-based enzymatic assay

(bottom). **b**, Enzymatic activity of purified hCEPT1 using CDP-choline as the substrate. Compared to the standard curve of CDP-choline (left), a CMP peak appeared when WT enzyme was added (middle), while no CMP peak was observed when inactive enzymes were added (right). The CMP peak was validated by MS (middle, inset). **c**, Enzymatic activity of purified hCEPT1 using CDP-ethanolamine as the substrate. Compared to the standard curve of CDP-ethanolamine (left), a CMP peak appeared when WT enzyme was added (middle), while no CMP peak was observed when inactive enzymes were added (right). The CMP peak was validated by MS (middle, inset). **d**, The HPLC curve and MS profile (inset) of the standard sample for CMP. This result is used as a standard to identify the peak position of CMP in the enzymatic assay shown in **b** and **c**. **e**, Enzymatic comparison of hCEPT1 using CDP-choline and CDP-ethanolamine as the substrates. Data are the mean ± s.d. of three independent experiments. **f**. Measurement of the binding affinity between hCEPT1 and CDP-choline using isothermal titration calorimetry.

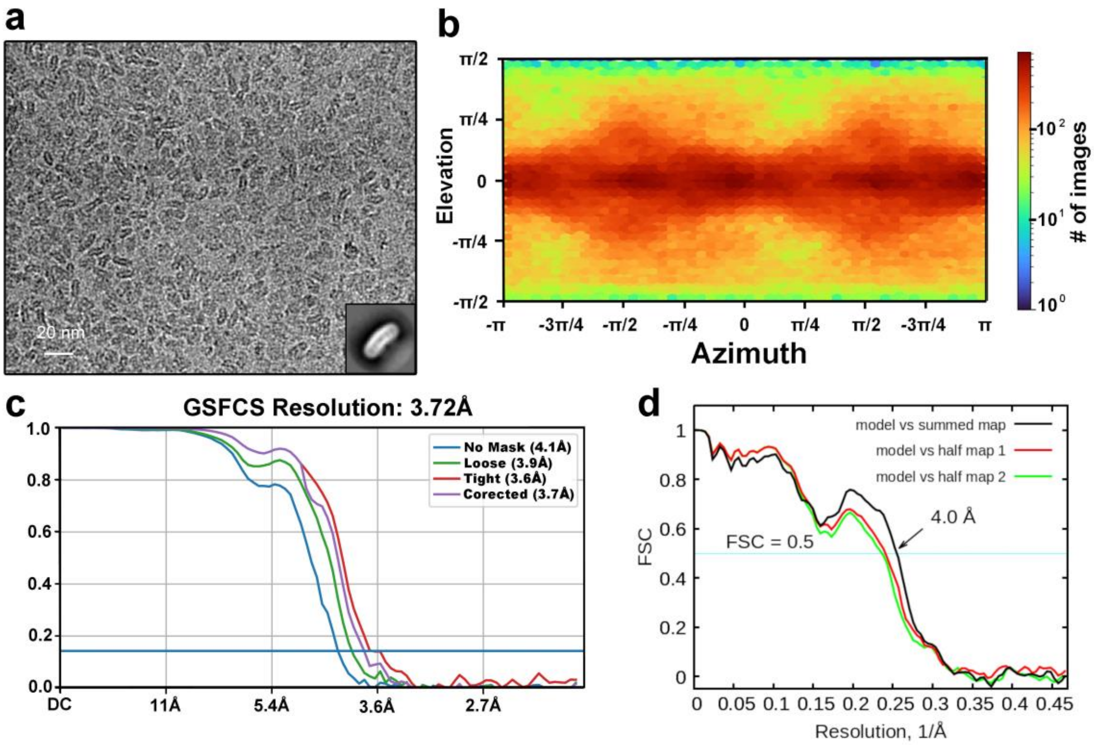

Supplementary Figure 2 | Cryo-EM structural analysis of human CEPT1. a, A representative micrograph and a 2D class average (inset) of cryo-samples of CEPT1 in GDN micelles. The box size for 2D averages is 214 Å. Scale bar, 20 nm. **b,** Angular distribution map for the final reconstruction. **c,** Golden standard Fourier Shell Correlation (GSFSC) curve for the final reconstruction. **d,** FSC curves of the refined model versus the summed map that it was refined against (black); of the model refined in the first of the two independent maps used for the gold-standard FSC versus that same map (red); and of the model refined in the first of the two independent maps versus the second independent map (green) for the final reconstruction.

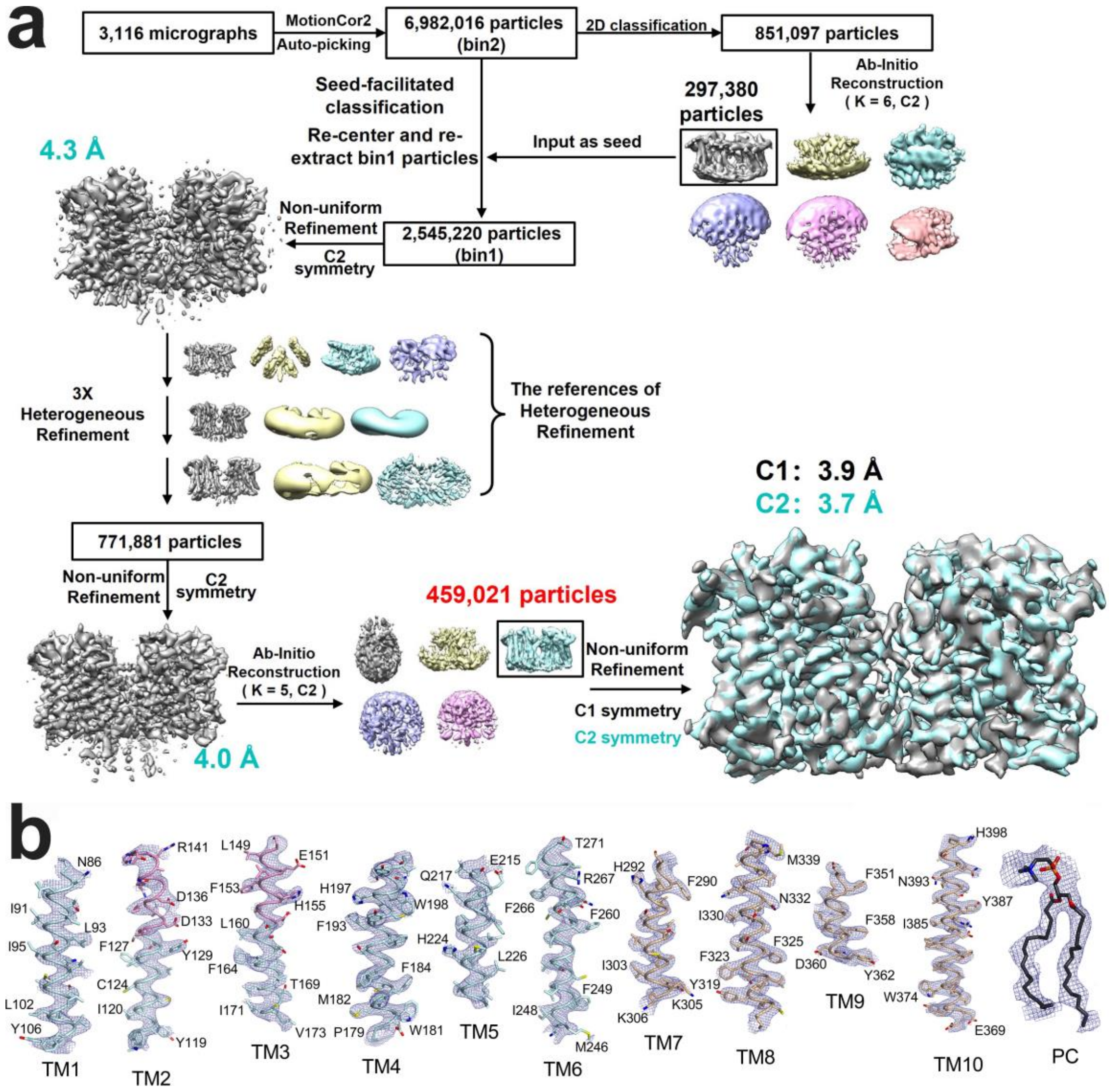

Supplementary Figure 3 | Flowchart for the structural determination of CEPT1.

a, Flowchart of data processing. Details are provided in the Materials and Methods. **b**, EM maps of representative structural elements. The densities, contoured at 7 σ, were prepared in PyMol.

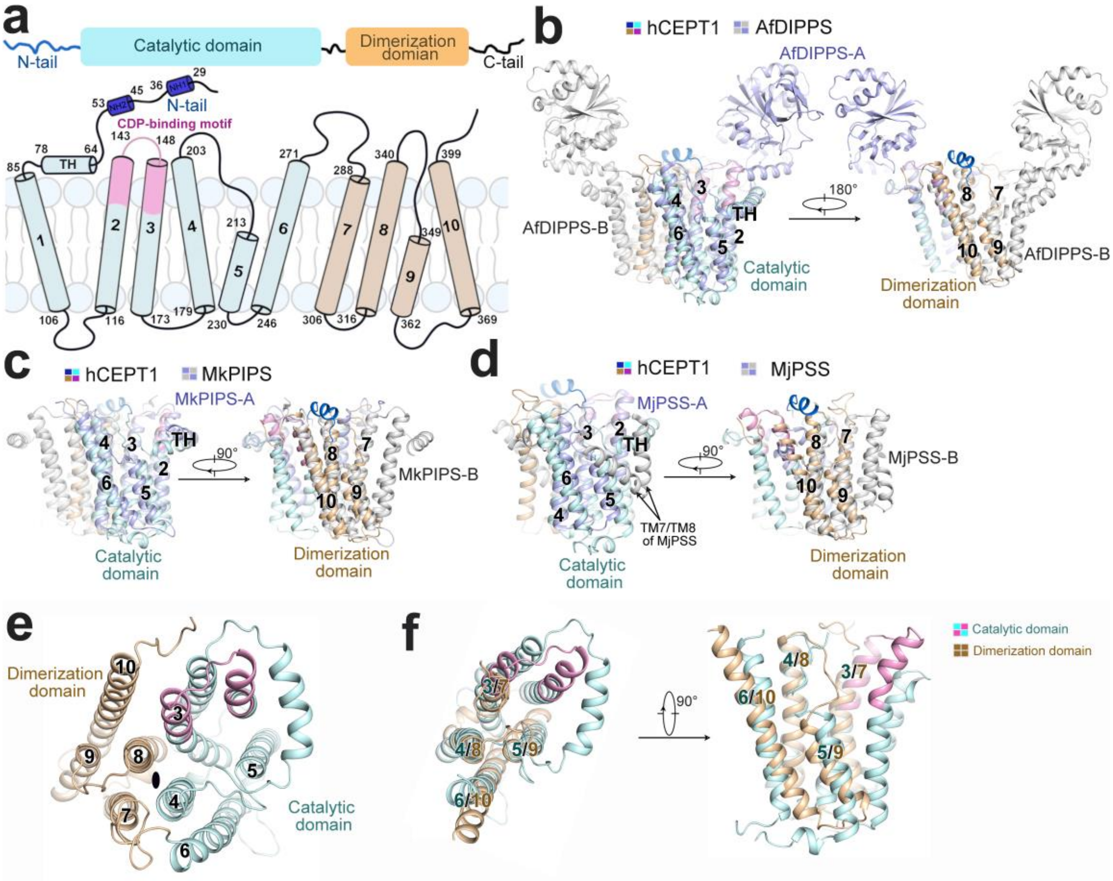

Supplementary Figure 4 | A conserved catalytic domain consists of TH and TM1-6. a, Domain organization (upper) and membrane topology (bottom) of CEPT1 based on our structure. **b-d,** Structural comparisons of hCEPT1 with AfDIPPS (PDB code: 4MND), MkPIPS (PDB code: 6WM5), and MjPSS (PDB code: 7B1K). AfDIPPS, MkPIPS, and MjPSS are all dimers with two protomers (A and B). The catalytic domain and dimerization domain of CEPT1 are superimposed with protomers A (left) and B (right), respectively. AfDIPPS, di-myo-inositol-1,3′-phosphate-1′-phosphate synthase from *Archaeoglobus fulgidu*s. MkPIPS, phosphatidylinositol phosphate

synthase from *Mycobacterium kansasii*. MjPSS, phosphatidylserine synthase from *Methanocaldococcus jannaschii*. **e,** The C2 pseudosymmetry axis is indicated by a black oval at the interface between the catalytic domain and dimerization domain. **f,** Superimposition of the dimerization domain with the catalytic domain of CEPT1 is shown in two perpendicular views.

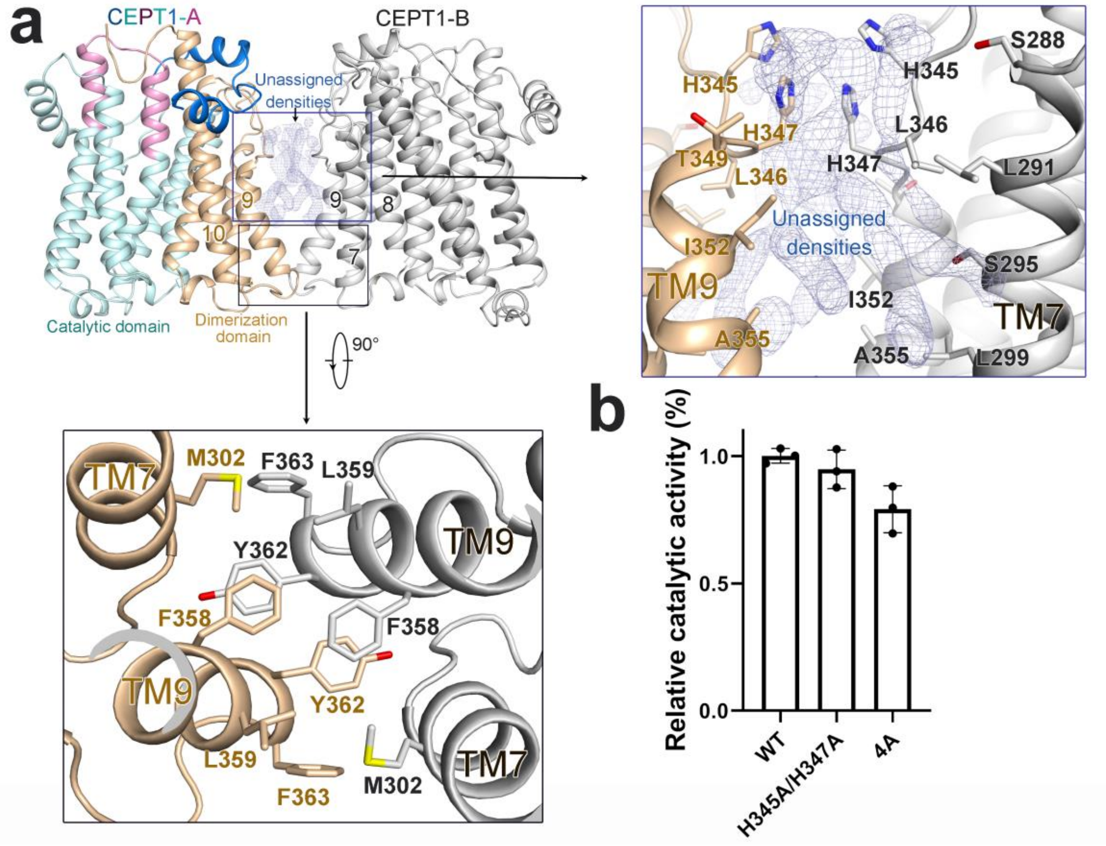

Supplementary Figure 5 | Dimeric organization of CEPT1. a, The dimeric interface of CEPT1. Protomer A is domain-colored, and protomer B is color gray. The

unassigned densities between two protomers are contoured at approximately 4.5 σ. The insets show residues on the dimeric interface. **b,** The enzymatic activities were not influenced when mutations were introduced to the dimeric interface. 4A, F358A/L359A/Y362A/F363A. Data in **b** are the mean ± s.d. of three independent experiments.

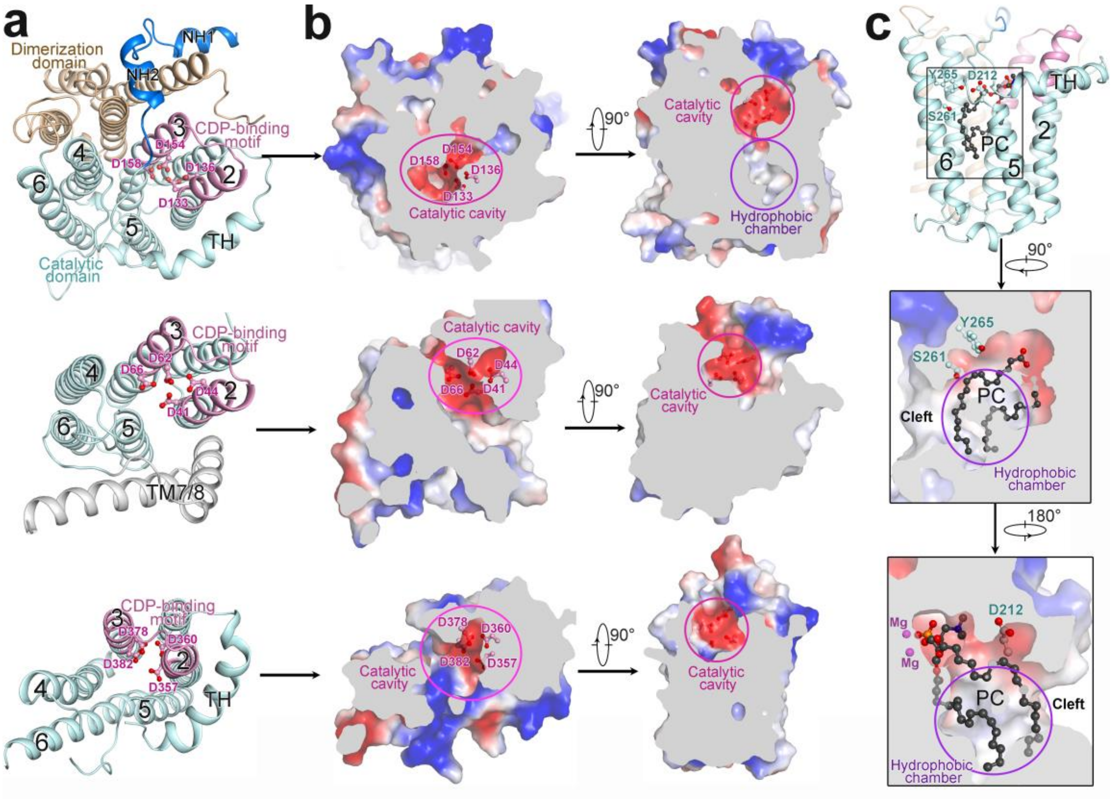

Supplementary Figure 6 | The hydrophobic chamber is unique in hCEPT1 compared to its bacterial/archaeal homologs. a, Cytosolic view of the structures for hCEPT1 (upper), MjPSS (middle, PDB code: 7B1K), and AfDIPPS (bottom, PDB code: 4MND). MjPSS and AfDIPPS are domain-colored with the same color code as CEPT1. The extra TMs 7 and 8 of MjPSS are colored gray. **b**, The electrostatic surface potentials of hCEPT1 (upper), MjPSS (middle), and AfDIPPS (bottom) are shown in two perpendicular views. The catalytic cavities are labeled with red circles, and the unique hydrophobic chamber is selected with a purple circle. **c**, Three polar residues (Asp212, Ser261, and Tyr265) are observed on the top of the hydrophobic

chamber. Insets: endogenous PC (colored black) in the hydrophobic chamber is shown in two opposite views.

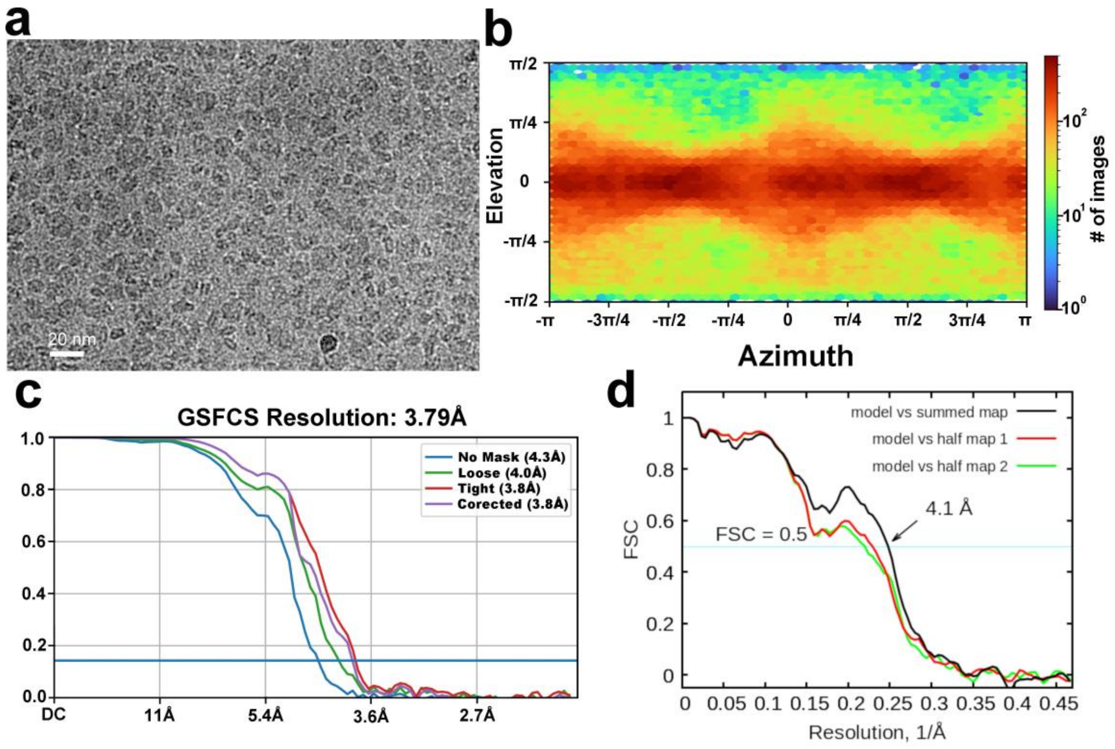

Supplementary Figure 7 | Cryo-EM structural analysis of the CEPT1 complex with CDP-choline. a, A representative micrograph of cryo-samples of CEPT1 complexed with CDP-choline. Scale bar, 20 nm. **b,** Angular distribution map for the final reconstruction. **c,** Golden standard Fourier Shell Correlation (GSFSC) curve for the final reconstruction. **d,** FSC curves of the refined model versus the summed map that it was refined against (black); of the model refined in the first of the two independent maps used for the gold-standard FSC versus that same map (red); and of the model refined in the first of the two independent maps versus the second independent map (green) for the final reconstruction.

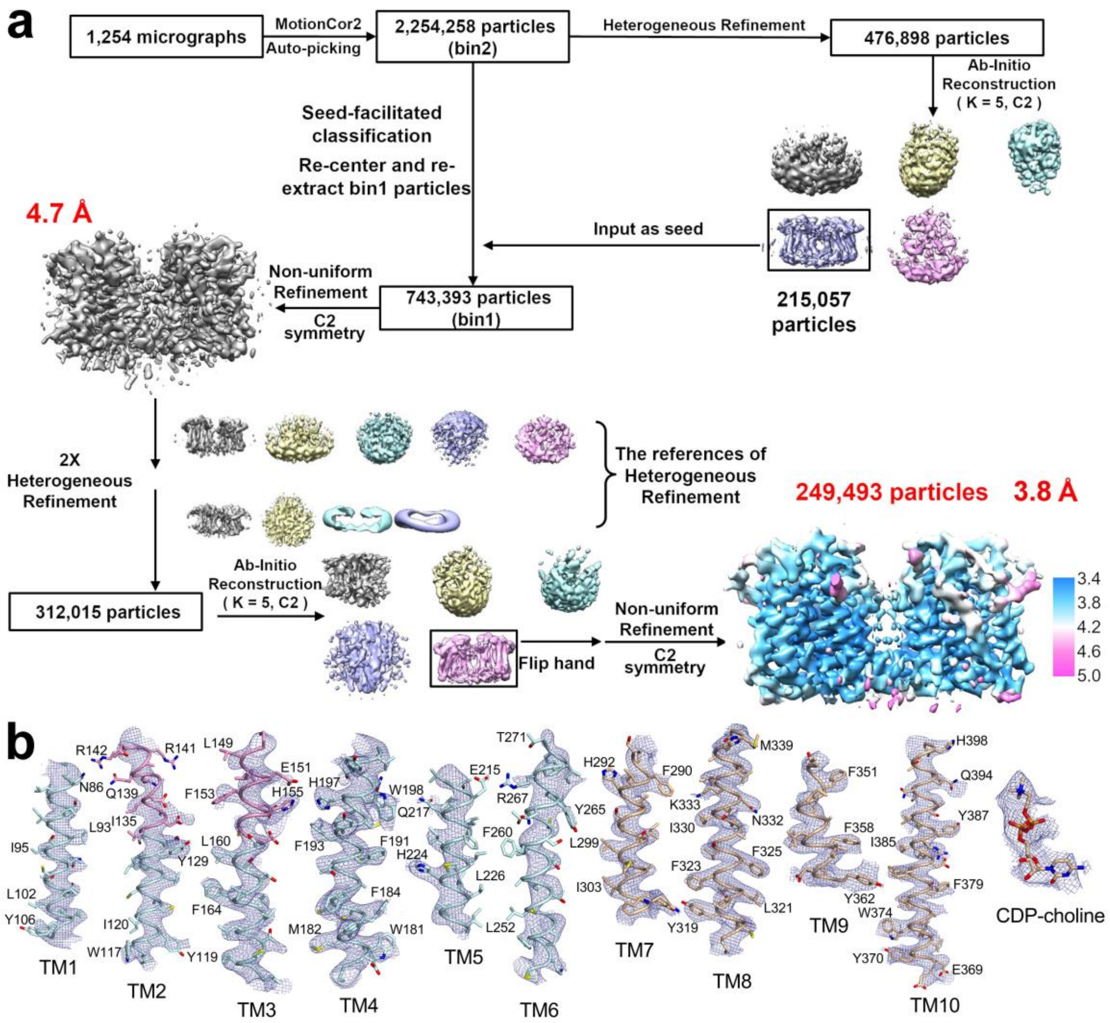

Supplementary Figure 8 | Flowchart for the structural determination of CEPT1 complexed with CDP-choline. a, Flowchart of data processing. Details are provided in the Materials and Methods. **b**, EM maps of representative structural elements. The densities, contoured at 7 σ, were prepared in PyMol.

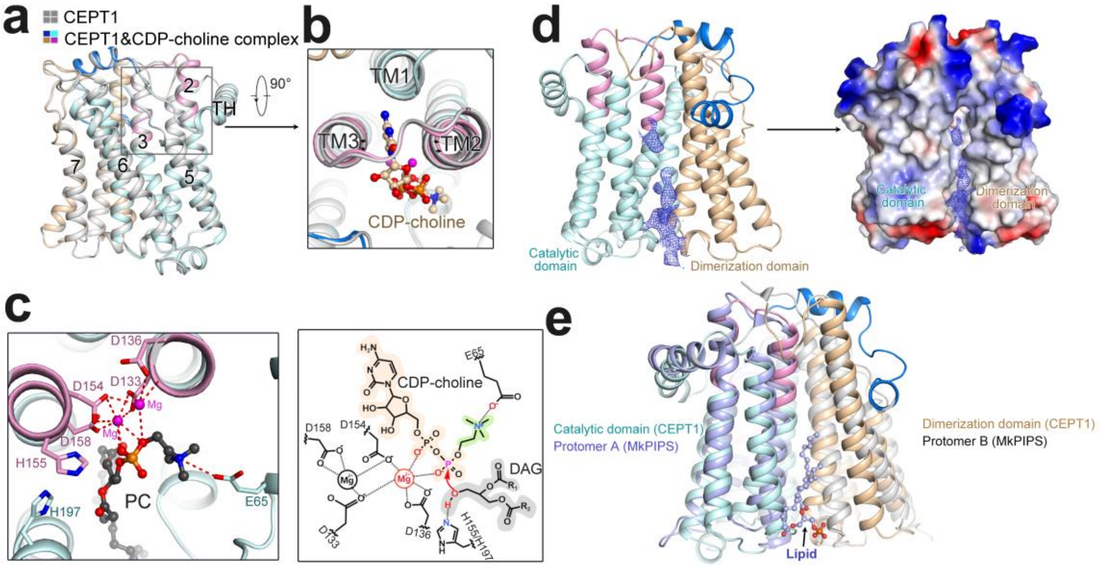

Supplementary Figure 9 | The proposed working mechanism during the catalytic process. a, Superimposition of the structure of CEPT1 and its complex with CDP-choline. **b,** The cytidine group is sandwiched between TM2 and TM3. The minor shifts of TM2 and TM3 are indicated with black arrows. **c**, Proposed catalytic mechanism. Left: His155 and His197 are close to the catalytic cavity. Right: His155 or His197 deprotonate the 3-OH group of DAG to facilitate nucleophilic attack toward the β-phosphorus atom of CDP-choline. **d,** Discontinuous densities**,** contoured at 4 σ, are observed at the hydrophobic groove between the catalytic domain and dimerization domain. **e,** A phospholipid is coordinated at the dimeric interface of MkPIPS (PDB code: 6WM5). The position overlaps with the hydrophobic groove of CEPT1 shown in **c**. The phospholipid is shown as sticks and balls.

Supplementary Table 1 | Summary of data collection and model statistics.

	**CEPT1 (EMD-34379) (PDB 8GYX)**	**CEPT1 and CDP-choline complex (EMD-34378) (PDB 8GYW)**
**Data collection and processing**		
Magnification	81,000	81,000
Voltage (kV)	300	300
Electron exposure ($e^-/Å^2$)	50	50
Defocus range (μm)	-1.5 ~ -2.0	-1.5 ~ -2.0
Pixel size (Å)	1.07	1.07
Symmetry imposed	C2	C2
Initial particle images(no.)		
Final particle images (no.)	459,021	249,493
Resolution (Å)	3.7	3.8
FSC threshold	0.143	0.143
Map resolution range (Å)	3.2 ~ 4.0	3.4 ~ 5.0
**Refinement**		
Initial model (PDB code)	AlphaFold2	8GYX
Map sharpening B factor ($Å^2$)	270	275
Model composition		
Non-hydrogen atoms	6118	6076
Protein residues	760	760
Ligands	6	6
R.m.s. deviations		
Bond lengths (Å)	0.007	0.007
Bond angles (°)	1.36	1.46
Validation		
MolProbity score	1.34	1.37
Clashscore	6.23	6.64
Poor rotamers (%)	0.30	0.30
Ramachandran plot		
Favored (%)	99.21	99.21
Allowed (%)	0.79	0.79
Disallowed (%)	0	0

Supplementary Table 2 | List of primers used in this study.

Mutations	Sense primer (5'-3')	Antisense primer (5'-3')
CEPT1 WT	ATTTAGCGGCCGCATGAGTGGGCATCGATCAAC	CCGCTCGAGATGATGATTAGAATGAGCTGTAG
CEPT1 (E65A)	CTGCTTGCGCCCTTAATGCAAGGGTATTGG	CATTAAGGGCGCAAGCAGGGACCGTCCAGC
CEPT1 (N86A)	GCCCCAGCTCTCATCACCATCATTGGACTG	GGTGATGAGAGCTGGGGCAATCCAGGAGGG
CEPT1 (D136A)	GCTATTGCCGGGAAACAGGCAAGAAGAACC	CTGTTTCCCGGCAATAGCATCCAAAGACTG
CEPT1 (E151A)	CTGGGAGCACTTTTTGATCATGGCTGTGAT	ATCAAAAAGTGCTCCCAGAGGAGAACTACT
CEPT1 (D154A)	CTTTTTGCCCATGGCTGTGATTCACTATCA	ACAGCCATGGGCAAAAAGTTCTCCCAGAGG
CEPT1 (D158A)	GGCTGTGCCTCACTATCAACAGTTTTTGTG	TGATAGTGAGGCACAGCCATGATCAAAAAG
CEPT1 (T162F)	CTATCATTTGTTTTTGTGGTTCTTGGAACT	TCCAAGAACCACAAAAACAAATGATAGTGA
CEPT1 (T169N)	CTTGGAAATTGTATTGCAGTGCAGCTGGGG	TGCAATACAATTTCCAAGAACCACAAAAAC
CEPT1 (A196L)	TATTGTCTGCACTGGCAAACGTATGTTTCT	TTGCCAGTGCAGACAATAGAACATAAATGT
CEPT1 (D212N)	AACGTGACTGAAGTGCAAATCTTC	TTGCACTTCAGTCACGTTAATTATTCCAAATCG CAATGTTC
CEPT1 (V216L)	ACTGAACTGCAAATCTTCATAATAATCATG	GAAGATTTGCAGTTCAGTCACATCAATTAT
CEPT1 (Q217L)	GAAGTGCTAATCTTCATAATAATCATGCAT	TATGAAGATTAGCACTTCAGTCACATCAAT
CEPT1 (S261A)	ATATTTGCCTGTACAAATTACTTCCGTGTAAT	ATTTGTACAGGCAAATATGGTCCCTGCTACAG
CEPT1 (Y265F)	ACAAATTTCTTCCGTGTAATCTTCACAGGT	TACACGGAAGAAATTTGTACAGGAAAATATGG
CEPT1(H345A&H3 47A)	ATGGCCTTGGCCGACACAGCATTCATAGGTCC	TGTGTCGGCCAAGGCCATTTCACTTTTCGTCAT GTGT
CEPT1 (F358A& L359A &Y362A&F363A)	GCTGCGGACCAGGCTGCGAACAGCTTTATTGATG AATATATT	CGCAGCCTGGTCCGCAGCCAAAAGTGCCGGAC CTATGA

All the primers are synthesized at Beijing Tsingke Biotech Co., Ltd.
