# Supplementary material for: Structural basis for catalysis of human choline/ethanolamine phosphotransferase 1
Source: Nat Commun. 2023 May 3;14:2529. doi: 10.1038/s41467-023-38290-2 (PMC10156783; doi:10.1038/s41467-023-38290-2)
Supplement: Supplementary file 3 — Reporting Summary [file 41467_2023_38290_MOESM3_ESM.pdf]

nature portfolio

Corresponding author(s): Hongwu Qian
Last updated by author(s): Mar 26, 2023

Reporting Summary

Nature Portfolio wishes to improve the reproducibility of the work that we publish. This form provides structure for consistency and transparency in reporting. For further information on Nature Portfolio policies, see our Editorial Policies and the Editorial Policy Checklist.

Statistics

For all statistical analyses, confirm that the following items are present in the figure legend, table legend, main text, or Methods section.

n/a	Confirmed	
☐	☒	The exact sample size (*n*) for each experimental group/condition, given as a discrete number and unit of measurement
☐	☒	A statement on whether measurements were taken from distinct samples or whether the same sample was measured repeatedly
☒	☐	The statistical test(s) used AND whether they are one- or two-sided<br>*Only common tests should be described solely by name; describe more complex techniques in the Methods section.*
☒	☐	A description of all covariates tested
☒	☐	A description of any assumptions or corrections, such as tests of normality and adjustment for multiple comparisons
☐	☒	A full description of the statistical parameters including central tendency (e.g. means) or other basic estimates (e.g. regression coefficient) AND variation (e.g. standard deviation) or associated estimates of uncertainty (e.g. confidence intervals)
☒	☐	For null hypothesis testing, the test statistic (e.g. *F*, *t*, *r*) with confidence intervals, effect sizes, degrees of freedom and *P* value noted<br>*Give P values as exact values whenever suitable.*
☒	☐	For Bayesian analysis, information on the choice of priors and Markov chain Monte Carlo settings
☒	☐	For hierarchical and complex designs, identification of the appropriate level for tests and full reporting of outcomes
☒	☐	Estimates of effect sizes (e.g. Cohen's *d*, Pearson's *r*), indicating how they were calculated

Our web collection on statistics for biologists contains articles on many of the points above.

Software and code

Policy information about availability of computer code

Data collection	EPU(2.12)
Data analysis	RELION 3.0, MotionCor2 1.4.7, CTFFIND 4.1.8, Phenix 1.20, Coot 0.9.8, Pymol 4.6.0, Chimera 1.16, GraphPad Prism 8.0.2, cryoSPARC (v3.3.2)

For manuscripts utilizing custom algorithms or software that are central to the research but not yet described in published literature, software must be made available to editors and reviewers. We strongly encourage code deposition in a community repository (e.g. GitHub). See the Nature Portfolio guidelines for submitting code & software for further information.

Data

Policy information about availability of data

All manuscripts must include a data availability statement. This statement should provide the following information, where applicable:

- Accession codes, unique identifiers, or web links for publicly available datasets
- A description of any restrictions on data availability
- For clinical datasets or third party data, please ensure that the statement adheres to our policy

The atomic coordinates of CEPT1 and its complex with CDP-choline complexes have been deposited in the RCSB Protein Data Bank under the accession codes 8GYX (http://doi.org/10.2210/pdb8GYX/pdb) and 8GYW (http://doi.org/10.2210/pdb8GYW/pdb). The corresponding electron microscopy maps have been deposited in the Electron Microscopy Data Bank under the accession codes EMD-34379 (https://www.ebi.ac.uk/pdbe/entry/emdb/EMD-34379) and EMD-34378 (https://www.ebi.ac.uk/pdbe/entry/emdb/EMD-34378), respectively. The raw electron microscopy images used to build the 3D structure are available from the corresponding author (hongwuq@ustc.edu.cn) upon request. The paper makes use of RCSB Protein Data Bank accession codes 4MND (https://www.rcsb.org/

structure/4MND), 6WM5 (https://www.rcsb.org/structure/6WM5), and 7B1K (https://www.rcsb.org/structure/7B1K). Source data are provided with this paper.

Human research participants

Policy information about studies involving human research participants and Sex and Gender in Research.

Reporting on sex and gender	NONE
Population characteristics	NONE
Recruitment	NONE
Ethics oversight	NONE

Note that full information on the approval of the study protocol must also be provided in the manuscript.

Field-specific reporting

Please select the one below that is the best fit for your research. If you are not sure, read the appropriate sections before making your selection.

☒ Life sciences ☐ Behavioural & social sciences ☐ Ecological, evolutionary & environmental sciences

For a reference copy of the document with all sections, see nature.com/documents/nr-reporting-summary-flat.pdf

Life sciences study design

All studies must disclose on these points even when the disclosure is negative.

Sample size	No statistical methods were used to predetermine sample size. For the enzymatic activity assays, experiments were performed at least three independent times. The sample size was determined based on the reproducibility of the current recording. The data size of cryoEM was determined by the availability of the microscope time and the particle density on the grids.
Data exclusions	None
Replication	All experimental results were reproduced. All experiments were repeated at least three times.
Randomization	For structure refinement, all particles were randomly split into two groups. For the enzymatic activity assays, randomization is not relevant as no group allocation were performed.
Blinding	Investigators were not blinded to group allocation, because no grouping was needed.

Reporting for specific materials, systems and methods

We require information from authors about some types of materials, experimental systems and methods used in many studies. Here, indicate whether each material, system or method listed is relevant to your study. If you are not sure if a list item applies to your research, read the appropriate section before selecting a response.

Materials & experimental systems

n/a	Involved in the study	
☐	☒	Antibodies
☐	☒	Eukaryotic cell lines
☒	☐	Palaeontology and archaeology
☒	☐	Animals and other organisms
☒	☐	Clinical data
☒	☐	Dual use research of concern

Methods

n/a	Involved in the study	
☒	☐	ChIP-seq
☒	☐	Flow cytometry
☒	☐	MRI-based neuroimaging

Antibodies

Antibodies used	ANTI-FLAG M2 affinity gel (sigma, A2220). The gel was used for protein purification. The gel was packed at the bottom of a column and the cellular extractions were passed through the gel for more 5 times for efficient binding.

Validation	https://www.sigmaaldrich.cn/certificates/COFA/A2/A2220/A2220-BULK________SLCF5876__.pdf

Eukaryotic cell lines

Policy information about cell lines and Sex and Gender in Research

Cell line source(s)	HEK293F (Thermo Fisher Scientific, R79007)
Authentication	No further authentication was performed for commercially available cell lines.
Mycoplasma contamination	No tested for mycoplasma contamination.
Commonly misidentified lines (See ICLAC register)	No commonly misidentified cell lines were used.
